# Comparison of Patient Clinical characteristics and Outcomes Between Different COVID-19 Peak Periods: A Single Center Retrospective Propensity Matched Analysis

**DOI:** 10.7759/cureus.15777

**Published:** 2021-06-20

**Authors:** Simone A Jarrett, Kevin B Lo, Samir Shah, Martin Angelo Zanoria, Dahnish Valiani, Omotola O Balogun, Raul Hiedra, Zurab Azmaiparashvili, Gabriel Patarroyo Aponte

**Affiliations:** 1 Internal Medicine, Albert Einstein Medical Center, Philadelphia, USA; 2 Medicine, Sidney Kimmel College of Thomas Jefferson University, Philadelphia, USA; 3 Pulmonary and Critical Care and Sleep Medicine, Albert Einstein Medical Center, Philadelphia, USA

**Keywords:** novel coronavirus infection of 2019 (covid-19), corona virus disease, corona pandemic, sars-cov-2 (severe acute respiratory syndrome corona virus 2), corona virus pandemic, covid-19 (corona-virus disease)

## Abstract

Introduction

While Coronavirus disease 2019 (COVID-19) specific treatments have been instituted, overall mortality rates among hospitalized patients remain significant. Our study aimed to evaluate patient clinical characteristics and outcomes comparing the different COVID-19 infection peak periods.

Methods

This is a retrospective study of all adult patients hospitalized with a confirmed diagnosis of COVID-19 between March 1 to April 24, 2020 and November 1 to December 31, 2020, which corresponded to the first and second waves of COVID-19 infection in our institution, respectively. Demographic and clinical characteristics of the patients were compared and used for propensity matching. Clinical outcomes, such as need for intubation, renal replacement therapy and inpatient mortality were subsequently compared between the two groups.

Results

Patients in the second COVID-19 wave had a significantly higher body mass index (32.58 vs 29.83, p <0.001), as well as prevalence of asthma (14% vs 8%, p=0.019) and chronic kidney disease (42% vs 18%, p <0.001). Almost all patients in the second COVID-19 wave received corticosteroid treatment (99% vs 30%, p <0.001), and significantly more patients received remdesivir (43% vs 2%, p <0.001). Meanwhile, none of the patients in the second COVID-19 wave were treated with tocilizumab or hydroxychloroquine. Differences in clinical outcomes, such as need for renal replacement therapy or intubation, and median length of stay were not statistically significant. Inpatient mortality remained largely unchanged between the two COVID-19 peak periods.

Discussion/ Conclusion

In our institution, after propensity matched analysis, clinical outcomes such as need for renal replacement therapy, intubation and inpatient mortality remained unchanged between the two COVID-19 peak periods.

## Introduction

Coronavirus disease 2019 (COVID-19) is caused by a novel coronavirus, named severe acute respiratory syndrome coronavirus 2 (SARS-CoV-2). Originating in Wuhan, Hubei Province, Central China, the virus has made a global impact after being declared a pandemic on March 11, 2020 [1]. To this date, WHO has reported 136,996,364 confirmed cases of COVID-19, including 2,951,832 deaths [2].

COVID-19 may be asymptomatic, yet it can also present with severe pneumonia-like symptoms. Since the declaration of a global pandemic, it has wreaked havoc on healthcare systems throughout the globe [3]. Treatment modalities such as dexamethasone and remdesivir have already been recommended by international guidelines. However, mortality rates remain high [4]. Therefore, analyzing the similarities and differences between patients and clinical outcomes during the two peaks of the COVID-19 pandemic is essential to highlight ways to reduce morbidity and mortality associated with the infection.

This study aims to evaluate patient clinical characteristics and outcomes between the different COVID-19 peak periods and to assess trends and differences in management approaches during these different periods.

## Materials and methods

Patients and Methods

Study Design, Participants, and Data Collection

This study was a single-center retrospective analysis of all adult patients aged 18 years and above who were hospitalized with a confirmed diagnosis of COVID-19 via reverse transcriptase-polymerase chain reaction assay (RT-PCR) performed on nasopharyngeal swab specimens. Patients were excluded if they were discharged within 24 hours of admission, or were not hypoxemic (defined as oxygen saturation ≤ 94% while breathing ambient air). The periods chosen were from March 1 to April 24, 2020, and from November 1 to December 31, 2020, which corresponded to the first and second waves of COVID-19 infection in our institution, respectively. Demographic and clinical factors including age, gender, race, and medical comorbidities were extracted from electronic medical records with a standardized data collection form. COVID-19 specific treatments such as corticosteroids, tocilizumab, hydroxychloroquine, and remdesivir were recorded. Patterns of systemic and prophylactic anticoagulation were also extracted from the data. Clinical outcomes including the incidence of major bleeding or venous thromboembolism, need for intubation, vasopressor support, renal replacement therapy, inpatient mortality and length of stay were compared and analyzed. This study was approved by the institutional review board with modified protocol number: IRB-2020-436.

Statistical Analysis

Demographic and clinical variables were tabulated and presented using descriptive statistics and frequencies. Categorical variables were analyzed with chi-square testing. Normally distributed variables were presented using means and standard deviation, with an independent T-test used to find a significant difference between continuous variables. Skewed variables were analyzed using Mann Whitney U test and presented as medians and interquartile ranges. A significance level of 0.05 was used. Propensity matching was then done to help minimize the influence of selection bias and potential confounding. This was performed by using all the demographic and clinical variables as covariates with a one-to-one nearest neighbor matching algorithm at a caliper of 0.2. The standardized difference in means and distribution of propensity scores were used in assessing the improvement of covariate balance after propensity score matching (PSM). A standardized difference of less than the absolute value of 0.2 was taken to indicate the negligible difference in the mean or prevalence of a covariate between the compared groups [5]. All the above procedures were conducted with IBM SPSS Statistics (IBM SPSS Statistics for Windows, Version 23.0. IBM Corp., Armonk, NY) for Windows and SPSS PS Matching plug-in (Propensity score matching in SPSS, psmatching3.03, Felix Thoemmes, Cornell University/University of Tübingen).

## Results

**Figure 1:**
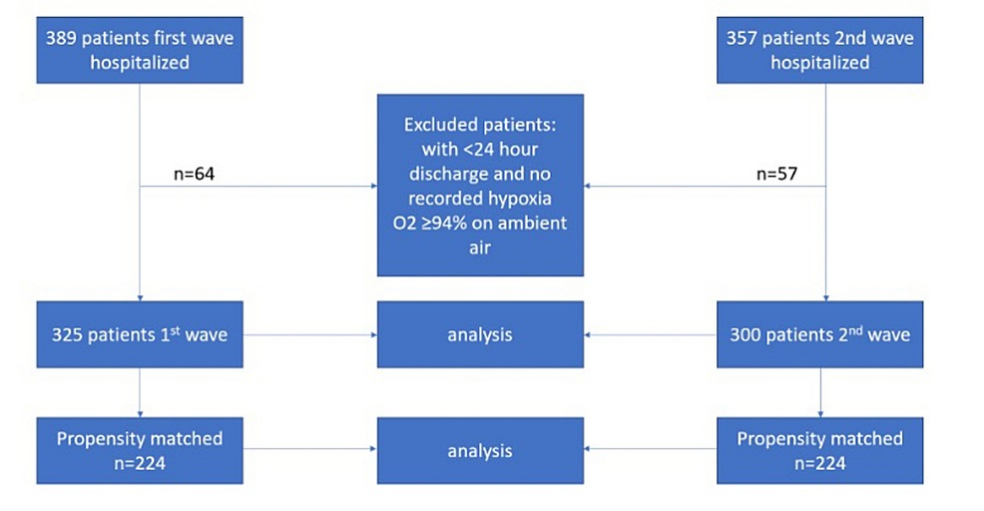
Propensity matching

A total of 389 and 357 patients were hospitalized during the first and second COVID-19 waves, respectively. Sixty four patients were excluded in the first wave, while 57 patients were excluded in the second wave based on defined exclusion criteria. A final sample of 625 patients (325 patients from the first wave, and 300 patients from the second wave, respectively) was used for final analysis. Propensity matching was done with a one-to-one matching adjustment for covariates, which yielded 224 patients per group (see figure 1).

In the unmatched cohort, 47% were females and 67% were African Americans, with the mean age of 65.66 ± 14.64 years. Patients in the second COVID-19 wave had a significantly higher BMI (32.58 vs 29.83, p <0.001), as well as prevalence of asthma (14% vs 8%, p 0.019) and chronic kidney disease (42% vs 18%, p <0.001). Almost all patients in the second COVID-19 wave received corticosteroid treatment (99% vs 30%, p <0.001), and significantly more patients received remdesivir (43% vs 2%, p <0.001). None of the patients in the second COVID-19 wave were treated with tocilizumab or hydroxychloroquine. In terms of patterns of anticoagulation use, significantly more patients in the second COVID-19 wave received prophylactic anticoagulation (74% vs 59% p<0.001) while none of the patients in the second COVID-19 wave received subtherapeutic doses of anticoagulation (please see Table 1 for demographic and clinical variables between the matched and unmatched cohorts). There were no significant differences in clinical outcomes, such as inpatient mortality, need for renal replacement therapy (RRT), intubation and length of stay. Inpatient mortality remained high at 23% vs 26%, p=0.512 (Table 2). This was despite the balance achieved among medical comorbidities between the two groups analyzed in the propensity matched cohort. 

**Table 1:** Clinical Characteristic of the Patients at Baseline comparing 1st and 2nd waves of COVID-19 in the unmatched and matched cohorts

Characteristics	First wave (n=325)	2^nd^ wave (n=300)	p value	First wave matched (n=224)	2^nd^ wave matched (n=224)	P value
Age mean ± SD	66.58±14.00	64.67±15.26	0.103	65.96±14.17	65.77±14.79	0.888
Female gender n (%)	162(50)	130(43)	0.109	110(49)	106(47)	0.777
Ethnicity n (%) African American Caucasian Hispanic Other	221(68) 41(13) 29(9) 34(11)	196(65) 34(11) 41(14) 29(10)	0.307	143(64) 31(14) 24(11) 26(12)	151(67) 26(12) 25(11) 22(10)	0.799
Comorbidities						
BMI (mean±SD)	29.83±9.01	32.58±10.99	<0.001	30.49±9.48	30.74±8.22	0.770
COPD	42(13)	49(16)	0.257	29(13)	32(14)	0.689
Asthma	25(8)	41(14)	0.019	25(11)	22(10)	0.758
Heart Failure	56(17)	55(18)	0.754	43(19)	39(17)	0.714
Atrial fibrillation	35(11)	29(10)	0.693	27(12)	22(10)	0.545
Liver cirrhosis	10(3)	5(2)	0.302	6(3)	4(2)	0.751
Diabetes	150(46)	128(43)	0.421	102(46)	99(44)	0.849
Chronic kidney disease all stages	58(18)	127(42)	<0.001	53(24)	65(29)	0.238
End stage renal disease on dialysis	38(12)	27(8)	0.296	25(11)	22(10)	0.758
Coronary artery disease	73(23)	55(18)	0.234	44(20)	42(19)	0.905
Hypertension	249(77)	224(75)	0.577	171(76)	171(76)	1.000
HIV	7(2)	7(2)	1.000	3(1)	5(2)	0.724
Medications used						
Antiplatelets	135(42)	108(36)	0.163	88(39)	81(36)	0.559
NOAC	27(8)	34(11)	0.226	23(10)	23(10)	1.000
Heparin lovenox	13(4)	9(3)	0.524	4(2)	2(1)	0.685
Warfarin	4(1)	12(4)	0.022	10(5)	6(3)	0.446
COVID-19 treatment						
Hydroxychloroquine	197(61)	0(0)	<0.001	131(59)	0(0)	<0.001
Steroids	96(30)	298(99)	<0.001	63(28)	222(99)	<0.001
Tocilizumab	41(13)	0(0)	<0.001	28(13)	0(0)	<0.001
Remdesivir	6(2)	128(43)	<0.001	4(2)	95(42)	<0.001
Anticoagulation regimen						
Prophylactic dose for VTE	192(59)	222(74)	<0.001	124(55)	172(77)	<0.001
Subtherapeutic dose	23(7)	0(0)	<0.001	14(6)	0(0)	<0.001
Therapeutic dose	97(30)	55(18)	0.001	72(32)	33(15)	<0.001
Venous thromboembolism (new)	30(9)	22(7)	0.469	19(9)	12(5)	0.264
GI bleeding	11(3)	6(2)	0.332	7(3)	4(2)	0.544
Brain bleed	3(1)	1(0.3)	0.625	2(1)	1(0.4)	1.000
Other site of bleeding	6(2)	8(3)	0.592	4(2)	7(3)	0.544

**Table 2:** Comparison of Clinical Outcomes in the unmatched and matched cohorts between the first and 2nd COVID-19 waves

Characteristics	First wave (n=325)	2^nd^ wave (n=300)	p value	First wave matched (n=224)	2^nd^ wave matched (n=224)	P value
Clinical outcomes						
Inpatient death	80(25)	79(26)	0.646	52(23)	59(26)	0.512
Need for CRRT/HD	55(17)	46(15)	0.664	36(16)	36(16)	1.000
Need for vasopressors	81(25)	66(22)	0.397	54(24)	52(23)	0.912
Need for intubation	89(27)	72(24)	0.360	60(27)	54(24)	0.588
Median length of stay (IQR)	8(4.5-14)	7(4-11)	0.015	7(4-14)	7(4-12)	0.250

## Discussion

As we already know with most illnesses, those with a history of multiple comorbidities tend to be predisposed to not only contracting the virus but also a more severe course of the disease [6]. In our population, it was found that more patients with a past medical history of asthma or chronic kidney disease were admitted to the hospital with COVID-19 during the second wave compared to the first peak. This is consistent with the previous studies showing a positive association between the presence of chronic kidney disease and the risk for hospitalization and poorer clinical outcomes in the setting of COVID-19 infection [7]. On the other hand, while there has been much debate in the literature regarding asthma as a risk factor for COVID-19, a recent meta-analysis did not find supportive evidence to suggest that asthma was associated with a higher risk for severe illness or increased mortality associated with COVID-19 infection [8]. The higher prevalence of asthma in patients admitted to the hospital with the diagnosis of COVID-19 infection may be a product of selection bias or a precautionary measure due to perceived concerns for poorer outcomes. Another significant finding from our study was that a higher proportion of patients with obesity were admitted to the hospital during the second wave of COVID-19 infection. This is not surprising and in line with the evidence linking obesity to a higher risk for severe COVID-19 infection and thereby, higher admission rates [9].

Early in the COVID-19 pandemic, largely driven by urgency and positive results in viral load reduction, as shown by clinical studies, hydroxychloroquine was granted FDA approval for the treatment of COVID-19 [10]. Similarly, due to positive outcomes with regards to attenuating the cytokine storm, relieving clinical symptoms, producing radiologic improvement, and halting progression to acute respiratory distress syndrome (ARDS) in the setting of COVID-19 infection, tocilizumab was also actively used as a treatment modality [11]. During the second wave of the COVID-19 pandemic, however, a trend of increased use of remdesivir and corticosteroid therapy was detected, due to having more available and robust evidence [12,13]. While hydroxychloroquine has fallen out of favor, tocilizumab has recently been recommended by international guidelines for the treatment of patients with progressive COVID-19 infection but has been adopted less in our institution during the second infection peak [14]. However, the matched cohort analysis showed that even after balancing the differences in comorbidities, the clinical outcomes of interest remained the same. A possible explanation to this is that healthcare systems may remain under pressure especially during COVID-19 surge periods. A recent study study of over 955 hospitals showed significantly varying mortality rates across different US hospitals with increases in county-level case rates (such as the COVID-19 peaks in this study) were associated with worsening risk-standardized event rate of hospital mortality and referral to hospice [15]. While the continued high mortality rates observed during the 2nd peak of COVID-19 in our study could reflect differences in quality of care [16], it could also mean differences in admission thresholds across hospitals and the highly heterogenous manner of severity or presentation of the COVID-19 disease entity itself [15].

Another potential explanation is that perhaps the true benefits of guideline recommended medications are diminished in a real-world setting considering the number of factors involved outside the environment of rigorously planned randomized controlled trials. For example, in our study, all patients required supplemental oxygen due to evidence of hypoxia on presentation, and although the mortality benefit of dexamethasone was stronger among those who required intubation in the RECOVERY trial, the overall mortality benefit remained modest at best [12]. In addition, in the clinical trials studying remdesivir, the use of the drug only shortened disease duration but did not have a significant effect on mortality [13]. Also, of importance was that virtually all patients in the second wave of the pandemic were placed on prophylactic doses of anticoagulation for the prevention of venous thromboembolism. Before this, there was some evidence to suggest that the use of subtherapeutic or therapeutic doses of anticoagulation may improve clinical outcomes. However, more recent studies have shown the risk of possible harm, including the risk for major bleeding with the indiscriminate use of anticoagulation in the treatment of COVID-19 infection [17,18]. Based on these findings and the data collected from our study, it appears that the best supportive care or guideline-directed management of complications such as ARDS and venous thromboembolism remain important cornerstones in the management of COVID-19.

Limitations

This study is limited within itself by its design of a retrospective (non-randomized) single-center analysis. Despite being within the same year, because the two groups compared are from different periods, there may be other potential confounding factors that can influence clinical outcomes. Nevertheless, this study presents a real-world view of the impact of COVID-19 infection in a community-based inner-city population setting and tries to elicit differences in patient profile and practices in the management of COVID-19. Of note, this study had a significant population size of over 600 patients and the use of propensity matching helped to account for a proportion of confounding. The generalizability of this study, however, is limited due to the preponderance of African Americans and other ethnic minorities with Caucasians representing the minority, comprising just 12% of the total population. We could not comment further on some clinical outcomes, such as VTE and major bleeding, due to the few event rates.

## Conclusions

In our institution, after propensity matched analysis, clinical outcomes such as need for renal replacement therapy, intubation and inpatient mortality remained unchanged between the two COVID-19 peak periods.
